# Relapsing-Remitting Perimyocarditis and Emerging Acute Myeloid Leukemia

**DOI:** 10.1016/j.jaccas.2026.109741

**Published:** 2026-08-07

**Authors:** Madeline B. Zipperer-Giblin, John I. Bowman, Nidhi Patel, Debbie Rinde-Hoffman

**Affiliations:** a Department of Internal Medicine, University of South Florida, Morsani College of Medicine, Tampa, Florida, USA; b Division of Cardiovascular Sciences, Department of Internal Medicine, University of South Florida, Morsani College of Medicine, Tampa, Florida, USA; c Heart and Vascular Institute, Tampa General Hospital, Tampa, Florida, USA

**Keywords:** acute heart failure, cancer, cardiac magnetic resonance, reduced ejection fraction

## Abstract

**Background:**

Perimyocarditis as an initial manifestation of acute myeloid leukemia (AML) is rare, with fewer than 5 contemporary cases reported.

**Case Summary:**

A 66-year-old man experienced 7 hospitalizations over 3 months for fever, elevated troponin, and inflammatory markers after Coxsackievirus B exposure. Initial cardiac magnetic resonance (CMR), infectious, and hematologic work-ups were unremarkable. Repeat CMR revealed biventricular dysfunction, new lateral wall late gadolinium enhancement, myocardial edema, and a circumferential pericardial effusion. Endomyocardial biopsy confirmed severe lymphocytic myocarditis. The patient failed 2 corticosteroid tapers but improved with colchicine, intravenous immunoglobulin, and anakinra. After cardiac recovery, peripheral blood and bone marrow evaluation revealed AML.

**Discussion:**

This case highlights a potential rare dual-etiology mechanism for perimyocarditis involving viral injury and AML-driven inflammatory activation.

**Take-Home Message:**

This case underscores the diagnostic value of serial CMR with biopsy, the role of interleukin-1 blockade and intravenous immunoglobulin in steroid refractory disease, and vigilance for emerging hematologic malignancy.

## Visual Summary

**Figure:**
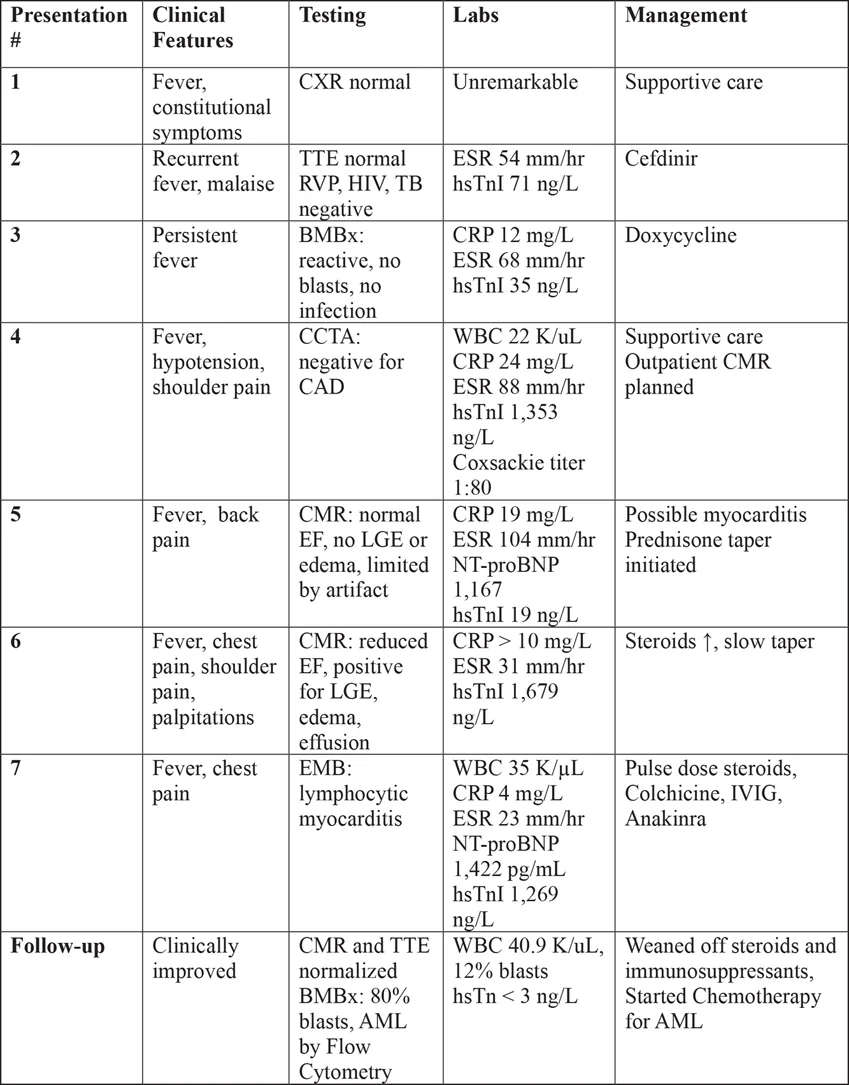

## History of Presentation

A 66-year-old man presented with fever, chills, night sweats, and shoulder and back pain after exposure to a grandchild with hand-foot-and-mouth disease. During his initial hospitalization, physical examination results were unremarkable.

**Take-Home Message**

• The case underscores the diagnostic utility of serial cardiac magnetic resonance with endomyocardial biopsy in refractory presentations, the application of 2025 European Society of Cardiology criteria to stage diagnosis across hospitalizations, the therapeutic rationale for IL-1 blockade and intravenous immunoglobulin in steroid-refractory perimyocarditis, and the important consideration of underlying hematologic malignancy driving steroid-refractory perimyocarditis.

## Past Medical History

The past medical history included hypertension, remote colon cancer status post resection, obstructive sleep apnea, and iron deficiency anemia.

## Differential Diagnosis

The differential diagnosis at his initial hospitalization included unspecified viral illness, which evolved to include coronary artery disease, heart failure, myocarditis, infection, and malignancy.

## Investigations

During his first emergency department visit (Figure 1), chest radiography, respiratory viral panel, magnesium level, comprehensive metabolic panel, and complete blood count results were unremarkable except for mild leukocytosis of 13.63 K/μL. Inflammatory markers and electrocardiography (ECG) were not obtained. He was discharged with supportive care for suspected viral illness. He returned 2 weeks later with recurrent fever and malaise. The high-sensitivity troponin I (hsTnI) level was mildly elevated at 71 ng/L (upper reference limit, 52 ng/L). ECG showed sinus tachycardia and left anterior fascicular block without significant ST-segment changes. Transthoracic echocardiography revealed an ejection fraction of 60% to 65% and no regional wall motion abnormalities. An extensive infectious work-up, including blood cultures, respiratory viral panel, HIV, QuantiFERON-TB, fungal serologies, and cryptococcal antigen, was negative. The troponin level normalized, and he was discharged on cefdinir. The troponin level elevation was suspected to be supply-demand mismatch related to infection.

**Figure 1:**
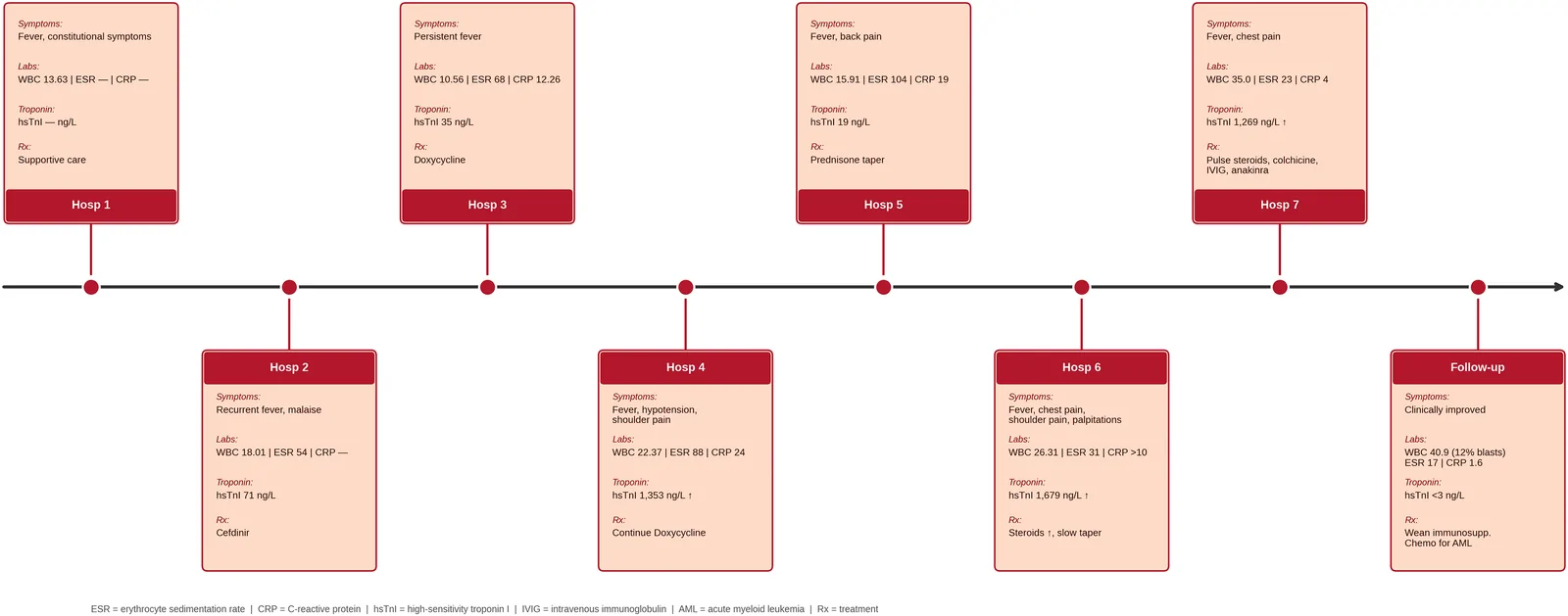
Timeline of Hospitalizations The patient had 7 hospitalizations over the course of 4 months. This timeline describes the patient's hospitalizations including symptoms present, WBC count, ESR, CRP, troponin, and the subsequent treatment. WBC = white blood cell.

He returned the following week with persistent fever. Laboratory tests showed a C-reactive protein level of 12 mg/L, an erythrocyte sedimentation rate of 68 mm/h, and an hsTnI level of 35 ng/L. ECG was unchanged. Extended infectious work-up, including Q fever, *Bartonella*, *Ehrlichia*, *Babesia*, Lyme, and Histoplasmosis, was negative. Bone marrow biopsy showed hypercellular marrow, myeloid predominance, and left-shift with normal flow cytometry, chromosome analysis, cytogenetics, and fluorescence in situ hybridization. He showed clinical improvement and was discharged on doxycycline.

One week later, he returned with fever, left shoulder pain, dizziness, and hypotension. The hsTnI level was 1,353 ng/L. The white blood cell count was 22.37K/μL, the C-reactive protein level was 23.72 mg/L, and the erythrocyte sedimentation rate was 88 mm/h. ECG showed sinus tachycardia with borderline ST-segment elevation laterally. Coronary computed tomography angiography excluded significant epicardial coronary artery disease. Coxsackie B IgG serology returned weakly positive at 1:80 (reference interval <1:10), suggestive of current or past infection. Transthoracic echocardiography revealed an ejection fraction of 60% to 65% without regional wall motion abnormalities or pericardial effusion. Inpatient cardiac magnetic resonance (CMR) was deferred because of patient preference, and he was discharged with instructions to complete doxycycline.

He was admitted the following week with continued fever. ECG revealed sinus tachycardia with a prolonged QTc and ischemic changes. CMR demonstrated normal left ventricular size and preserved systolic function (left ventricular ejection fraction 59%) with no myocardial edema on T2-weighted sequences. There was no obvious late gadolinium enhancement (LGE), though evaluation was limited by artifact. In the absence of definitive CMR or endomyocardial biopsy (EMB) findings, this presentation was classified as possible myocarditis per 2025 European Society of Cardiology (ESC) criteria.^1^ He was discharged on a 6-week prednisone taper (initial dose 40 mg daily).

## Management

After weaning prednisone to 0.25 mg/kg, the patient developed chest pain and fever, and he was readmitted. Repeat CMR (3 weeks after initial CMR) showed a left ventricular ejection fraction of 41%, a right ventricular ejection fraction of 44%, new LGE in the basal-to-mid lateral wall, myocardial edema on T2-weighted sequences, and a small circumferential pericardial effusion (Figure 2). This constellation met criteria for definitive perimyocarditis per 2025 ESC guidelines, and prednisone was increased to 0.5 mg/kg. Guideline-directed medical therapy was initiated with sacubitril-valsartan and metoprolol succinate, and he was discharged with a plan for close outpatient monitoring.

**Figure 2:**
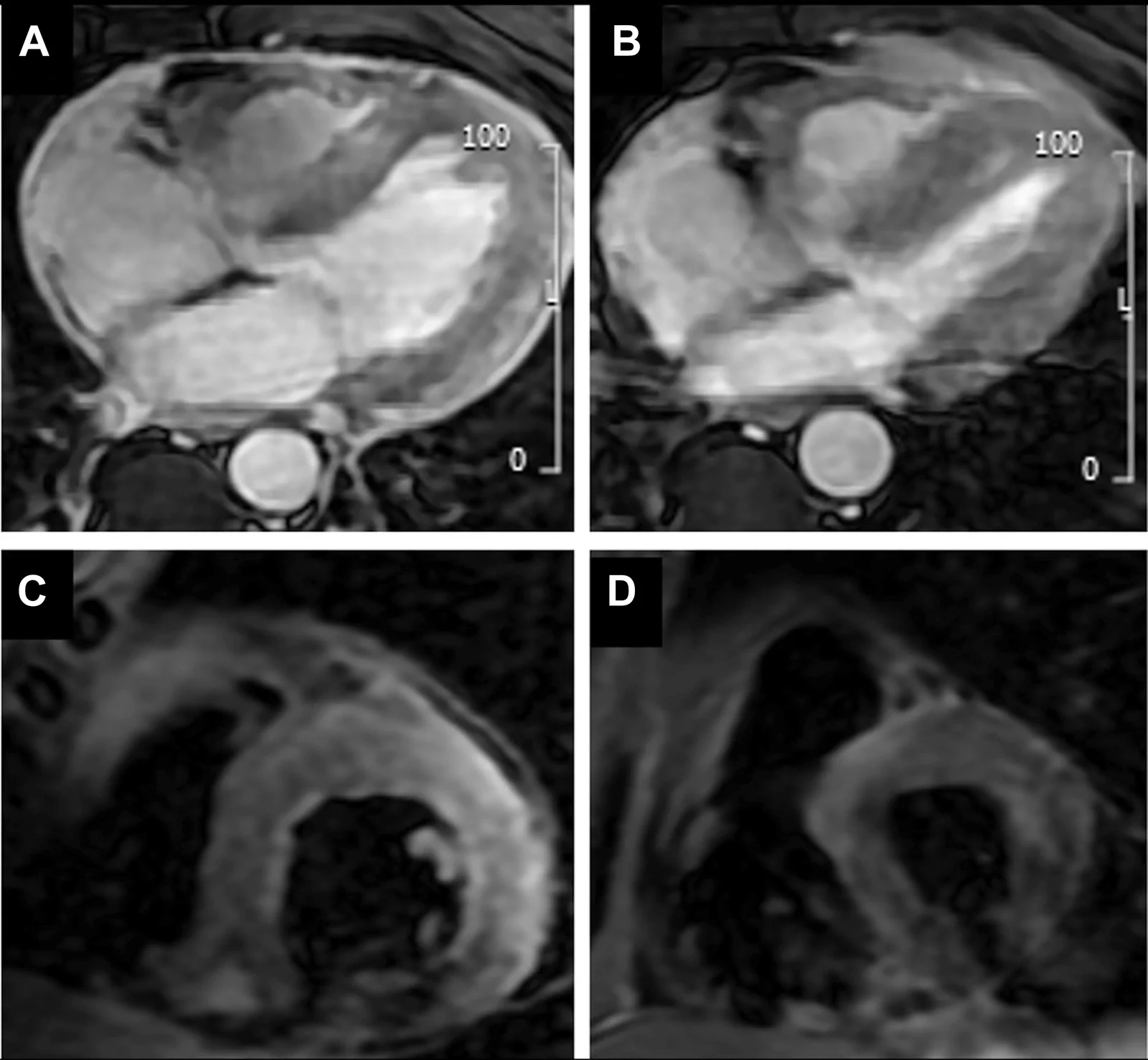
Cardiac Magnetic Resonance Showing Pericardial Enhancement and Myocardial Edema Cardiac magnetic resonance on the left during hospitalization 7 showing circumferential pericardial enhancement (A) and myocardial edema predominantly in the lateral wall (C). Cardiac magnetic resonance on follow-up 2 weeks later showing resolution of pericardial enhancement (B) and resolution of myocardial edema (D).

He was readmitted with recurrent symptoms after a second failed prednisone taper (0.25 mg/kg/d). Given 2 episodes of corticosteroid failure, EMB was performed, and pulse-dose steroids were initiated. EMB confirmed severe myocarditis with a predominantly lymphocytic infiltrate and no evidence of giant cells, eosinophilic infiltrate, or granulomas—histologic pattern most consistent with viral-mediated injury (Figure 3). Given the now-definitive diagnosis of steroid-refractory perimyocarditis, escalation to pericarditis-directed immunomodulatory therapy was initiated with colchicine 0.6 mg twice daily, anakinra 100 mg subcutaneous daily, and intravenous immunoglobulin (IVIG) (2 doses, 1,000 mg/kg).

**Figure 3:**
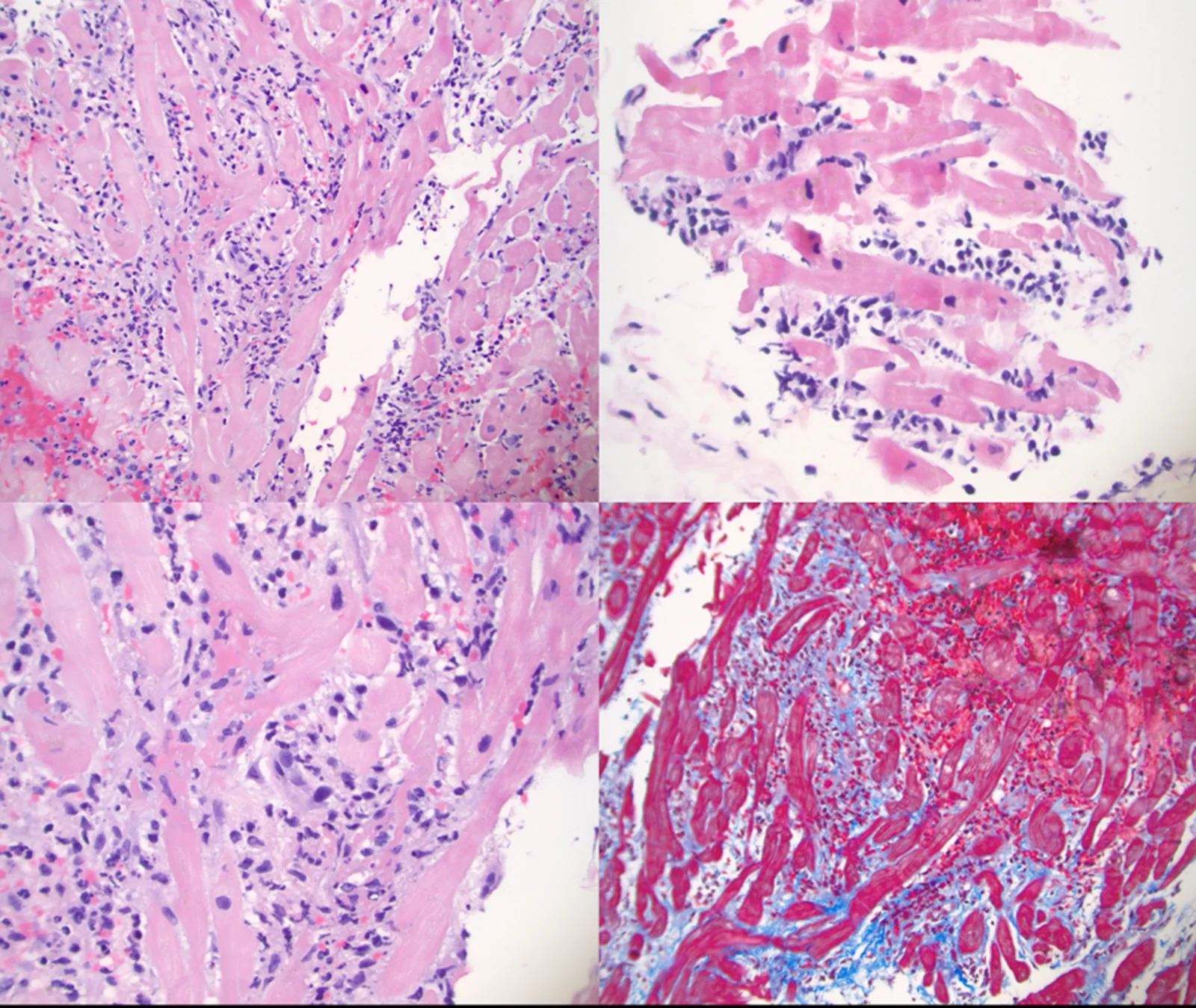
Endomyocardial Biopsy Demonstrating Severe Lymphocytic Myocarditis Histopathology reveals dense predominantly lymphocytic inflammatory infiltrates with associated myocyte injury. Admixed neutrophils are present (a nonspecific finding that may reflect severe tissue injury). There was no evidence of giant cells, eosinophilic predominance, or granulomatous inflammation. Background features include granulation tissue formation and interstitial fibrosis (confirmed by trichrome stain), consistent with subacute to chronic evolution. Ancillary studies are negative for adenovirus, cytomegalovirus, Epstein-Barr virus (in situ hybridization), parvovirus, and fungal organisms (Grocott–Gomori’s Methenamine Silver stain).

## Outcome and Follow-Up

Follow-up CMR 3 days after these new therapies demonstrated normalization of left ventricular function and resolution of LGE, myocardial edema, and pericardial effusion (Figure 2). Troponin and inflammatory markers normalized. Prednisone and anakinra were subsequently weaned and discontinued. However, routine follow-up complete blood count revealed a markedly elevated white blood cell count of 40.9K/μL with 12.2% peripheral blasts. Repeat bone marrow biopsy demonstrated 80% blasts with flow cytometry consistent with acute myeloid leukemia (AML). The patient was referred to hematology and initiated chemotherapy with cladribine, cytarabine, and venetoclax.

## Discussion

This case presents a 66-year-old man with 7 hospitalizations over 3 months for a relapsing-remitting perimyocarditis culminating in a diagnosis of AML, one of the rarest reported associations in the cardiology literature.^2^, ^3^, ^4^, ^5^ Although 3 of the 4 reported cases were diagnosed with AML at the time of myopericarditis diagnosis, Haboub et al^2^ reported a case of myopericarditis preceding a diagnosis of AML by 5 months in a 16-year-old girl, a timeline similar to our case. Our case extends this observation to an older adult and demonstrates dual viral and hematologic contributors, an association not previously described.

After exposure to Coxsackievirus, infection of cardiomyocytes leads to cell damage and death, and innate immune cells are activated through pathogen-associated molecular patterns and damage-associated molecular patterns.^6^ Pattern recognition receptors activate immune cells to produce and release proinflammatory and antiviral cytokines. Intracellular and surface antigens are released from the heart due to the cytolytic effects of Coxsackie that then stimulate T and B lymphocytes, leading to the activation of cytotoxic T lymphocytes to kill infected cells. Over time, activation of the innate immune system can lead to chronic inflammation, myocardial destruction, remodeling, and dysfunction.^6^ The patient's EMB revealed severe myocarditis with predominantly lymphocytic infiltrates, suggesting viral etiology.

However, the patient's eventual diagnosis of AML also raises the question whether underlying inflammation due to emerging hematologic malignancy contributed to the patient's relapsing-remitting course. Overexpression of tumor necrosis factor, activation of the nuclear factor-κB pathway, and increased production of interleukin (IL)-1β have been identified in the pathogenesis of AML and lead to overproduction of inflammatory cytokines. A similar overproduction of cytokines and amplification of the inflammatory response have been implicated in the pathogenesis of acute myocarditis.^7^

This case required progressive escalation through 3 therapeutic tiers over multiple hospitalizations. Corticosteroids were initiated as first-line immunosuppressive therapy after the diagnosis of possible myocarditis. Two separate taper attempts failed, prompting escalation. Steroid failure in myocarditis is not uncommon in the setting of ongoing viral antigen exposure or concurrent inflammatory amplification from a secondary process such as AML, reflecting the complexity of potential dual-mechanism disease.^8^

The 2025 ESC guidelines strongly recommend colchicine for acute and recurrent pericarditis.^1^ Although its role in pure myocarditis is less established, the pericardial involvement in this case provided a clear indication. Colchicine inhibits neutrophil chemotaxis, nod-like receptor protein 3 inflammasome assembly, and IL-1β release, mechanistic targets relevant to both pericarditis and the AML-driven inflammatory milieu.^9^

IVIG is a well-established therapy in selected myocarditis presentations. Its mechanisms include Fc receptor saturation reducing antibody-mediated cytotoxicity, cytokine neutralization, complement inhibition, and regulatory T-cell expansion. The 2024 American College of Cardiology Expert Consensus Decision Pathway acknowledges IVIG as a reasonable option in refractory or fulminant myocarditis, particularly when immune-mediated pathology is suspected.^8^ In this case, the lymphocytic infiltrate and steroid resistance supported its use. IVIG for pericarditis is less frequently studied but has been used in steroid-dependent or colchicine-resistant pericarditis.

Anakinra, a recombinant human IL-1 receptor antagonist, has emerged as the most evidence-supported biologic for recurrent and refractory pericarditis. The Anakinra-Treatment of Recurrent Idiopathic Pericarditis (AIRTRIP) trial demonstrated that anakinra significantly reduced recurrence rates compared with placebo in patients with colchicine-resistant idiopathic recurrent pericarditis.^10^ The 2025 ESC guidelines now recommend anakinra as a class IIa indication for colchicine-resistant recurrent pericarditis.^1^ Anakinra interrupts the NLRP3/IL-1 axis central to both autoinflammatory pericarditis and, notably, AML-driven cytokine production.

The favorable CMR response observed in this case after addition of anakinra and IVIG supports therapeutic rationale, though causality cannot be established from a single case. The temporal response to IVIG and anakinra supports consideration of early IL-1 blockade in patients with recurrent or steroid-refractory perimyocarditis, particularly when pericardial inflammation is a prominent feature. This case also raises important questions about the attribution of perimyocarditis in the context of concurrent infectious and neoplastic stimuli. We propose that both mechanisms were likely operative: Coxsackievirus B provided the initial cardiomyocyte injury and lymphocytic infiltration, whereas emerging AML amplified and perpetuated the inflammatory cascade, explaining the severity, refractoriness, and relapsing course.

## Conclusions

We present a rare case of relapsing-remitting perimyocarditis with a proposed dual-etiology framework involving Coxsackievirus B–mediated autoimmune injury and concurrent emerging AML-associated cytokine dysregulation. Cases of perimyocarditis in the setting of AML remain exceedingly rare in the literature. Multidisciplinary collaboration between cardiologists, hematologists, and basic scientists is essential to define optimal treatment strategies and improve outcomes in this complex patient population.

**Visual Summary:** Clinical Timeline, Diagnostic Findings, and Management

Presentation	Clinical Features	Testing	Laboratory Tests	Management
1	Fever, constitutional symptoms	CXR normal	Unremarkable	Supportive care
2	Recurrent fever, malaise	TTE normal; RVP, HIV, and TB negative	ESR 54 mm/h, hsTnI 71 ng/L	Cefdinir
3	Persistent fever	BMBx: reactive, no blasts, no infection	CRP 12 mg/L, ESR 68 mm/h, hsTnI 35 ng/L	Doxycycline
4	Fever, hypotension, shoulder pain	CCTA: negative for CAD	WBC 22K/μL, CRP 24 mg/L, ESR 88 mm/h, hsTnI 1,353 ng/L, Coxsackie titer 1:80	Supportive care, outpatient CMR planned
5	Fever, back pain	CMR: normal EF, no LGE or edema, limited by artifact	CRP 19 mg/L, ESR 104 mm/h, NT-proBNP 1,167 pg/mL, hsTnI 19 ng/L	Possible myocarditis, prednisone taper initiated
6	Fever, chest pain, shoulder pain, palpitations	CMR: reduced EF, positive for LGE, edema, effusion	CRP >10 mg/L, ESR 31 mm/h, hsTnI 1,679 ng/L	Steroids ↑, slow taper
7	Fever, chest pain	EMB: lymphocytic myocarditis	WBC 35K/μL, CRP 4 mg/L, ESR 23 mm/h, NT-proBNP 1,422 pg/mL, hsTnI 1,269 ng/L	Pulse dose steroids, colchicine, IVIG, anakinra
Follow-up	Clinically improved	CMR and TTE normalized; BMBx: 80% blasts, AML by flow cytometry	WBC 40.9K/μL, 12% blasts, hsTn <3 ng/L	Weaned off steroids and immunosuppressants, started chemotherapy for AML

AML = acute myeloid leukemia; BMBx = bone marrow biopsy; CAD = coronary artery disease; CCTA = coronary computed tomography angiography; CMR = cardiac magnetic resonance; CRP = C-reactive protein; CXR = chest x-ray; EF = ejection fraction; EMB = endomyocardial biopsy; ESR = erythrocyte sedimentation rate; hsTnI = high-sensitivity troponin I; IVIG = intravenous immunoglobulin; LGE = late gadolinium enhancement; NT-proBNP = N-terminal pro–B-type natriuretic peptide; RVP = respiratory viral panel; TB = tuberculosis; TTE = transthoracic echocardiography; WBC = white blood cell.

## Funding Support and Author Disclosures

This study was funded by the Tampa General Hospital's Heart and Vascular Institute. The authors have reported that they have no relationships relevant to the contents of this paper to disclose.
